# Infrastructural and human-resource factors associated with return of infant HIV test results to caregivers: secondary analysis of a nationally representative situational assessment, South Africa, 2010

**DOI:** 10.1186/s12879-019-4337-0

**Published:** 2019-09-16

**Authors:** Nobubelo Kwanele Ngandu, Vincent Maduna, Gayle Sherman, Nobuntu Noveve, Witness Chirinda, Vundli Ramokolo, Carl Lombard, Ameena Ebrahim Goga

**Affiliations:** 1 Health Systems Research Unit, South, African Medical Research Council, Cape Town, SA South Africa; 2 Centre for HIV and STI, National Institute of Communicable Diseases, Johannesburg, SA South Africa; 3 Department of Paediatrics and Child Health, Faculty of Health Sciences, University of Witwatersrand, Johannesburg, SA South Africa; 4 Biostatistics Unit, South African Medical Research Council, Cape Town, SA South Africa; 5 School of Public Health and Family Medicine, University of Cape Town, Cape Town, SA South Africa; 6 Department of Paediatrics, University of Pretoria, Pretoria, SA South Africa

**Keywords:** Early infant diagnosis of HIV, Turn-around times, Laboratory transportation system, Human resources, SAPMTCTE, PMTCT

## Abstract

**Background:**

In June 2015, South Africa introduced early infant HIV diagnosis (EID) at birth and ten weeks postpartum. Guidelines recommended return of birth results within a week and ten weeks postpartum results within four weeks. Task shifting was also suggested to increase service coverage. This study aimed to understand factors affecting return of EID results to caregivers.

**Methods:**

Secondary analysis of data gathered from 571 public-sector primary health care facilities (PHCs) during a nationally representative situational assessment, was conducted. The assessment was performed one to three months prior to facility involvement in the 2010 evaluation of the South African programme to prevent mother-to-child HIV transmission (SAPMTCTE). Self-reported infrastructural and human resource EID-related data were collected from managers and designated staff using a structured questionnaire. The main outcome variable was ‘EID turn-around-time (TAT) to caregiver’ (caregiver TAT), measured as reported number of weeks from infant blood draw to caregiver receipt of results. This was dichotomized as either short (≤3 weeks) or delayed (> 3 weeks) caregiver TAT. Logit-based risk difference analysis was used to assess factors associated with short caregiver TAT. Analysis included TAT to facility (facility TAT), defined as reported number of weeks from infant blood draw to facility receipt of results.

**Results:**

Overall, 26.3% of the 571 PHCs reported short caregiver TAT. In adjusted analyses, short caregiver TAT was less achieved when facility TAT was > 7 days (versus ≤7 days) (adjusted risk difference (aRD): − 0.2 (95% confidence interval − 0.3-(− 0.1)), *p* = 0.006 for 8–14 days and − 0.3 (− 0.5-(− 0.1)), p = 0.006 for > 14 days), and in facilities with staff nurses (compared to those without) (aRD: − 9.4 (− 16.6-(− 2.2), *p* = 0.011).

**Conclusion:**

Although short caregiver TAT for EID was only reported in approximately 26% of facilities, these facilities demonstrate that achieving EID TAT of ≤3 weeks is possible, making timely ART initiation within 3 weeks of diagnosis feasible within the public health sector. Our adjusted analyses underpin the need for quick return of results to facilities. They also raise questions around staff mentoring: we hypothesise that facilities with staff nurses were likely to have fewer professional nurses, and thus inadequate senior support.

**Electronic supplementary material:**

The online version of this article (10.1186/s12879-019-4337-0) contains supplementary material, which is available to authorized users.

## Introduction

HIV/AIDS is still a major cause of infant morbidity and mortality in developing countries and is responsible for up to 20% of under-5 child deaths in South Africa [1, 2]. The main mode of HIV acquisition in infants is through mother-to–child transmission (MTCT). The effectiveness of programmes that prevent MTCT (PMTCT) can be improved through early diagnosis of HIV in mothers and infants, timely provision of maternal treatment and infant prophylaxis, expedited linkage to care and safe infant feeding practices postnatally. Early infant diagnosis of HIV (EID) using HIV polymerase chain reaction (PCR) is critical for appropriately managing HIV infected or HIV exposed uninfected infants. To be effective in reducing morbidity and mortality in HIV infected infants, EID needs to be complemented with timely return of results to the caregiver and timely initiation of triple antiretroviral treatment (ART) [3]. The time period between infant HIV testing and receipt of results by the caregiver should be kept as short as possible to avoid delayed ART initiation in HIV infected infants.

The challenge of achieving and maintaining short turnaround times (TAT) for EID for timely ART initiation is widespread in low and some middle income countries, despite the adoption of test and treat and life-long ART. For example, a 2016 review of EID turnaround times in Lesotho demonstrated a median TAT of more than 60 days from blood draw to return of results to caregivers [4]. In general, reasons for poor TAT are multidimensional [5]. The distances and efficiency of transportation systems between various service providers along the EID pipeline play an important role in determining the TAT from blood draw to return of results to caregivers in most less-resourced countries [6, 7]. A cohort study following up HIV-exposed infants from two till eighteen months of age in rural Kenya examined the dynamics of EID and revealed poor human resource capacity as one of the barriers [8]. Another qualitative study which interviewed a sample of caregivers, community leaders and healthcare workers from three provinces in Mozambique reported service provider attitudes, overcrowding, unreliable transportation of specimens to the laboratory and delayed blood test results as inhibitors to EID services [9]. Task shifting, defined as delegating tasks to staff with lower level qualifications has been proposed as a solution to increase the efficiency HIV-related service coverage. A systematic review demonstrated that although task shifting may be cost-effective and may improve care, careful implementation is needed, including adequate and sustainable training and support for staff in new roles [10].

The EID TAT for South Africa are not well reported in the literature. At initial PMTCT roll-out, the recommended timing for routine EID was 6 weeks post-partum, with return of results within four weeks. However, in June 2015, HIV PCR testing at birth, with return of results within one week, was introduced to identify intra-uterine infections, which have a poorer prognosis, and initiate infant ART as early as possible [11]. However, if EID TAT are delayed, then the intention underpinning these revisions will be jeopardized. As point-of-care technologies for EID have not been widely adopted in South Africa; it is essential to understand TAT. This paper aims to understand factors affecting EID TAT to caregivers (caregiver TAT), and specifically asks whether service provider-related infrastructural and human resource factors are associated with caregiver TAT.

## Methods

### Study design and data collection

A cross-sectional study design was used to conduct a nationally-representative situational assessment in 571 primary health care (PHC) facilities conducting EID activities. This occurred, one to three months prior to their involvement in the 2010 nationally representative survey to evaluate the effectiveness of the South African PMTCT program (SAPMTCTE). Details of the situational assessment have been reported elsewhere [12]. Briefly, facilities were the primary sampling units and selection occurred with probability proportional to size. Facilities were stratified by province and annual immunisation workload of less than 130 immunizations, between 130 and 300 and over 300. The latter group was further stratified into antenatal HIV prevalence greater than 29% or less than or equal to 29%. The final stratification according to size was; large1 (> 300 annual immunizations and > 29% antenatal HIV prevalence), large2 (> 300 annual immunizations and ≤ 29% antenatal HIV prevalence), medium (130–300 annual immunizations) and small (< 130 annual immunizations). There were 35 strata in total across provinces. The desired number of facilities were randomly selected within each stratum.

Non-clinical data collectors were trained for four days prior to data collection. Structured questionnaires were used to collect data from clinic managers, nurses providing immunization, PMTCT nurses, nurses providing sick child (including Integrated Management of Childhood Illnesses (IMCI)) services and district health information officers. Interviews were conducted using open and closed-ended questions. Human resource data were gathered on the total number of personnel available per facility, number trained to perform counselling and blood draw for EID using nationally accredited standard operating procedures (SOP) and number of staff members not trained in the SOP for EID blood draw but performing EID counselling and blood draw. Infrastructural questions included transportation of specimens to the laboratory, type of facility which was either a clinic or community health center (CHC), availability of supplies and EID TAT. One delegated staff member answered each section of the questionnaire. Data relating to EID TAT were reported by a senior staff member working on postnatal care with access to information about specimen submission to the laboratory, receipt of results from the laboratory, and return of results to the caregiver. Two questions were asked, one on average TAT for return of results to the facility (facility TAT) and the other on average TAT for return of results to mothers (caregiver TAT), from day of infant blood draw. Staff members were encouraged to check records to answer these questions, and to reflect on and draw from their overall experience. Only one questionnaire was answered per facility. The questionnaire was piloted in two health facilities in the Western Cape prior to the commencement of the study.

At the time of the study, national protocols only allowed professional nurses (not staff nurses) to perform infant blood draw for EID. A designated transport system (laboratory-run or private courier) collected the dried blood spots (DBS) from facilities and transported to the nearest of 14 HIV PCR reference laboratories. Facilities and laboratories were situated at variable distances away from one another. Following analysis and authorization at the reference laboratory, hard copy results were printed, sorted and returned to the facility using the same designated transport system as before. All six weeks PCR test results were expected to be returned to caregivers by the 10 weeks routine visit but it was recommended that caregivers of HIV infected infants be contacted earlier.

### Ethical consideration

Ethics approval was obtained from the South African Medical Research Council Ethics Committee (Ref: EC09–002). Approval and buy-in were obtained from the National and Provincial Departments of Health prior to commencement of the study. Written consent was obtained from all participants.

### Data management

Data were captured on Excel and transferred to STATA v SE-14 for analysis [13].

The main outcome variable for this analysis was EID caregiver TAT, defined here as the time in weeks from date of infant blood draw to caregiver receipt of test results, as reported by health care providers. We present it as a binary outcome dichotomized as ‘short caregiver TAT’ of less than or equal to 3 weeks versus ‘delayed caregiver TAT’ of more than 3 weeks. We chose 3 weeks as the cut off for EID caregiver TAT, to test the feasibility of quick return of HIV-positive results, given the recommendations at the time as well as the current recommendations to return birth EID results within one week of testing and subsequent EID test results within 4 weeks of testing. Four infrastructural predictor variables were investigated for association with short caregiver TAT; (i) the type of transportation system used to transport infant blood samples between the health facility and the laboratory, categorized as either a provincially managed system, a system managed by the NHLS or a system tendered to a private courier; (ii) the size of the health care facility, small, medium or large, as defined in the sampling approach; (iii) the type of PHC, dichotomized as either a CHC or clinic; and (iv) the reported facility TAT, defined as total time taken for results to be returned to the health facility from date of blood draw, categorized as less than or equal to 7 days, 8–14 days and > 14 days, to accommodate the ability to achieve a one week or 3 weeks or 4+ weeks EID caregiver TAT. The time spent at the facility before the caregivers were informed of the infant HIV results was not available. Four human resource-related predictor variables were evaluated. These were binary variables each indicating whether a facility had staff nurses, enrolled nurse assistants, lay counsellors or doctors. In the descriptive analyses, we further expanded each staff type to show the proportion of those who were trained on the SOP for EID blood draw, or were already performing HIV testing activities. Nearly all facilities (~ 97%) had professional nurses and all the professional nurses were trained in SOP for EID blood draw and performed these activities, therefore it was not statistically feasible to include this staff type in the analyses. The non-doctor staff-type were defined as follows according to Woldesenbet, *et. al*. [14]: (i) professional nurses are registered nurses who have studied a four-year course and are legally authorized to practice by the examination board; (ii) staff nurses are those who completed a one year course at a recognized training institution (iii) enrolled nurse assistants are those who completed a one year course and assist senior nurses, and (iv) lay counsellors are members of the surrounding community who undergo short training and complement work done by registered nurses.

### Statistical methods

The prevalence of the outcome variable short caregiver TAT was calculated for each predictor variable of interest in bivariate tests, and a survey chi-squared *p*-value reported. Adjusted risk differences were used to determine the predictor variables associated with short caregiver TAT in three simple steps. In step 1, bivariate logistic regression models were constructed looking at each predictor variable independently. In step 2, the significant risk factors from step 1 (*p* value< 0.05) were all included into a multivariable logistic regression model. In step 3, the adjusted risk differences, presented as percentages, were calculated for each predictor variable using a post-test analysis from the model run in step 2. All percentages and statistics were weighted to adjust for the sampling design. A p value < 0.05 was taken to indicate a significant finding in all analyses.

## Results

### Descriptive analyses

Of the 571 PHCs sampled, only 26.3% (95% confidence interval (CI): 22.8–30.0%) reported short caregiver TAT, that is, in 26.3% of facilities, caregivers on average received their infants’ HIV results in 3 weeks or less from the time of infant blood draw (Table 1). Of the 571 facilities, 80 (14.6%) facilities used the provincially managed transportation system, 347 (59.5%) used the system managed by the NHLS and 110 (20.3%) tendered the transport to a private courier. Facility TAT of ≤7 days occurred in just above a quarter of the facilities (26.6%), a quarter (24.5%) had results returned in 8–14 days and 22.8% of facilities had results returned > 14 days.

**Table 1:** Distribution of predictor variables across all facilities and by short EID caregiver TAT

Predictor variables	All PHCs including those with short EID TAT, *N* = 571 N (%)	Short EID caregiver TAT (≤3 *weeks*) *N* = 150 Prevalence % [95% CI]	Chi-squared *P* value
Laboratory transportation system managed by:			**0.002**
Local Province	80 (14.6)	5.8 [2.9–11.2]	
NHLS	347 (59.5)	68.8 [61.1–75.6]	
Private courier	110 (20.3)	17.9 [12.6–24.7]	
Unknown	34 (5.7)	7.5 [4.3–12.9]	
Return of HIV results to facility (facility TAT)			**< 0.001**
≤ 7 days	152 (26.2)	48.3 [40.4–56.3]	
8–14 days	142 (24.5)	29.7 [22.9–37.6]	
> 14 days	130 (21.5)	5 [2.5–9.8]	
Unknown	147 (27.8)	17 [11.7–23.9]	
PHC Size			0.34
Large	268 (46.8)	46.4 [39.7–53.3]	
Medium	200 (34.8)	31.6 [25.5–38.4]	
Small	103 (18.4)	22 [17.1–27.7]	
Type of PHC			0.693
Community Health Center	42 (6.8)	6.1 [3.3–11.0]	
Clinic	529 (93.2)	93.9 [89.0–96.7]	
Facility with professional nurses			0.509
No	19 (3.2)	4.1 [1.9–8.7]	
Yes	552 (96.8)	95.9 [91.3–98.1]	
Facility with staff nurses			**0.007**
No	259 (43.2)	52.7 [44.9–60.4]	
Yes	312 (56.8)	47.3 [39.6–55.1]	
Facility with enrolled nurse assistants			0.223
No	154 (26.9)	30.7 [23.8–38.6]	
Yes	417 (73.1)	69.3 [61.4–76.2]	
Facility with lay counsellors			0.629
No	27 (4.8)	4.1 [1.8–8.7]	
Yes	544 (95.2)	95.9 [91.3–98.2]	
Facility with doctors			0.264
No	344 (60.4)	56.6 [48.7–64.2]	
Yes	227 (39.6)	43.4 [35.8–51.3]	
Available staff types trained on SOP for EID blood draw and/or performing EID activities			
Facility with staff nurses (*N* = 312)			0.783
No	214 (69.3)	70.7 [59.2–80.1]	
Yes	98 (30.7)	29.3 [19.9–40.8]	
Facility with enrolled nurse assistants (*N* = 417)			0.470
No	390 (93.8)	92.2 [85.1–96.1]	
Yes	27 (6.2)	7.8 [3.9–14.9]	
Facility with lay counsellors (*N* = 544)			**0.041**
No	498 (91.2)	95.6 [90.3–98.0]	
Yes	46 (8.8)	4.4 [2.0–9.7]	
Facility with doctors (*N* = 227)			0.393
No	181 (81.2)	84.8 [73.5–91.8]	
Yes	46 (18.8)	15.2 [8.2–26.5]	

*PHC* – primary health care facility. *TAT* – turnaround time, *EID*- early infant HIV diagnosis, 95% CI – 95% confidence intervals. *P*-values are from a survey chi-squared test. *NHLS*- National health laboratory services. *PCR*- polymerase chain reaction. *SOP*- standard operating procedures. The significance level used for the Chi-squared test is *p* value< 0.05, significant *p* values are in boldface

The reported distribution of different staff types varied widely: the majority of facilities had professional nurses (96.8%), enrolled nurse assistants (73.1%) and lay counsellors (95.2%). Staff nurses were reported in just above half of the sampled facilities while the support of doctors was reported in 39.6% of the facilities. All professional nurses were either trained on the SOP for EID blood draw or already engaged in EID activities. However, less than a third of each of the other staff types reported being trained on the SOP for EID blood draw or performing EID activities.

Of the 150 facilities with short EID caregiver TAT, most of their courier services (68.8%) were managed by the NHLS, 17.9% by a private courier and only 5.8% by the province; these prevalences were significantly different (*p* = 0.002, Table 1). Most facilities with short caregiver TAT had facility TAT of less than or equal to 7 days (48.3%), followed by facility TAT of 8–14 days (29.7%) while only 5% had results returned after 14 days (5.0%), *p* < 0.001). Caregiver TAT did not appear to differ significantly by facility size or by facility type. Among the human resource variables, short caregiver TAT was significantly lower among facilities with staff nurses (chi-squared *p* value = 0.007, although the 95% confidence intervals overlapped). There was no significant difference in caregiver TAT according to training on the SOP for EID blood draw or carrying out EID activities, per non-professional nurse staff types except in the case of lay counsellors. In all these non-professional nurse staff types however, whether significant or not, the prevalence of short EID caregiver TAT was very low in facilities with these non-professional nurse staff trained on the SOP for EID blood draw.

### Multivariable (adjusted) analysis of factors associated with short EID caregiver TAT

In adjusted risk difference analysis, short caregiver TAT was significantly associated with facility TAT and the staff nurses staff type (Table 2). Facilities with more than 7 days facility TAT reported achieving short caregiver TAT at 0.2 percentage points significantly less often than those who had results returned in ≤7 days, *p* = 0.006. The percentage points were reduced by 0.3 if the TAT to facilities was more than 14 days, p = 0.006. Facilities with staff nurses achieved short caregiver TAT at ~ 9 percentage points less often than facilities who did not have this staff type (*p* = 0.011) but the confidence interval for the adjusted risk difference was very wide (− 16.6-(− 2.2)). Although the prevalence of short caregiver TAT was very high among facilities supported by the NHLS transportation system, the absolute risk difference relative to provincial-supported facilities was not statistically significant (*p* = 0.254). However the data suggested that the NHLS-supported facilities had short caregiver TAT at 0.1 percentage points more often than the provincial-supported facilities.

**Table 2:** Adjusted Risk differences for having short EID caregiver TAT

	Adjusted RD (%)	95% CI	*P* value
Laboratory transportation system managed by:			
Local Province (Ref.)			
NHLS	0.1	−0.1 - 0.2	0.254
Private courier	0.2	−0.1 - 0.5	0.255
Return of HIV results to facility (facility TAT)			
≤ 7 days (Ref.)			
8–14 days	− 0.2	−0.3 - (− 0.1)	**0.006**
> 14 days	− 0.3	− 0.5 - (− 0.1)	**0.006**
Facility Size			
Large (Ref.)			
Medium	0.7	−4.0 - 5.4	0.762
Small	1.5	−8.1 - 11.0	0.764
Type of facility			
Community Health Center (Ref)			
Clinic	−5.6	−14.3 - 3.2	0.212
Facility with staff nurses			
No (Ref.)			
Yes	−9.4	−16.6 - (−2.2)	**0.011**
Facility with enrolled nurse assistants			
No (Ref.)			
Yes	−5.5	−13.4 - 2.3	0.169
Facility with lay counsellors			
No (Ref.)			
Yes	−0.2	−13.9 - 13.4	0.972
Facility with doctors			
No (Ref.)			
Yes	2.6	−4.6 - 9.9	0.478

*TAT* – turnaround time, *EID*- early infant HIV diagnosis, *RD*- Risk Difference, 95% CI – 95% confidence intervals. *PCR*- polymerase chain reaction. The significance level used for the Chi-squared test is *p* value < 0.05, significant *p* values are in boldface

## Discussion

Using data from a nationally representative situational assessment conducted in 2010, we demonstrate that a quarter of facilities managed to achieve short EID Caregiver TAT. Returning results to caregivers as soon as possible is important for additional HIV prevention counselling to ensure these infants remain negative. We assume that in the 26% of facilities that received test results within 3 weeks, infants were initiated on ART timeously and received early post-test interventions to optimize the health of caregivers and infants, in accordance with national policy. The study focused on caregiver TAT as an aggregated variable, and did not disentangle the steps within that process. In a Tanzania study by Manumbu *et. al*., time spent at each specific step of the EID process was monitored and the steps delaying the EID TAT were rectified, eventually leading to shorter EID caregiver TAT [15]. .

Delayed EID TATs, weak infrastructure as well as inadequate human resources, reportedly contribute to poor access to EID services in other settings [5]. In our study, we looked at factors associated with short EID caregiver TAT – from blood sample collection from an infant to return of results to the caregiver. EID caregiver TAT was not significantly associated with staff type other than staff nurses, training in SOPS for EID blood draw, facility size and facility type. The prevalence of short facility TAT was highest among facilities using the NHLS transportation system to the laboratory, with a significant chi-squared statistic for differences in prevalence between different transportation systems. This is understandable as the NHLS system comprised of several local laboratories servicing smaller areas than the provincial system, thereby facilitating better TAT. However, the adjusted risk difference was not statistically different because most facilities used the NHLS system, even most of those with delayed caregiver TAT of more than 3 weeks, giving little differential for comparison.

During this period (2010), the NHLS was in the process of taking over all transportation systems. The provincial transportation system was still being used by the KwaZulu Natal province at large (see Additional file 1) during this survey period. Although the other provinces were largely covered by the NHLS transport systems, some facilities used private couriers during this period. We observed the highest prevalence of short EID caregiver TAT outcomes with NHLS transport systems because since 2010, the NHLS began exploring and implementing new ways to reduce turnaround times for the routine healthcare services they offer to facilities and these included faster result return to the facilities by SMS printers [16]. As soon as an HIV PCR test was authorised in the laboratory, the result printed out in the facility so that healthcare workers did not have to wait for hard copy results to be printed at their local NHLS laboratory, sorted and transported to the facility before clinicians could take action on the results. Between then and now (2018), the NHLS has taken over all the urban EID transportation services for public healthcare facilities; however, support for remote rural facilities is not consistent. Because we did not see a statistically significant association with NHLS for short EID TAT, these findings rationalize the need to investigate the current TAT within the NHLS service alone. Most importantly, the performance of the service in remote and rural settings, for which no literature exists.

Time duration for return of HIV results to facility, i.e., TAT to facilities, was significantly associated with EID caregiver TAT. It is logical that the longer it takes to communicate between the facility and the laboratory, the longer it will take to return results to facility caregivers. This was true in this study. This association has also been observed in other African countries such as reported in a Uganda study published in 2013 [17]. However, our study has further confirmed that if the process between the facility and the laboratory takes more than 7 days, there is bound to be delays of at least 4 weeks in completing the EID process. Only a quarter of the facilities in this study sample achieved the 7 day time duration for return of HIV results to facility, which is a concerning observation. Wiegert et al., [18] in a study in Zimbabwe in 2012, demonstrated that 80% of the sampled facilities had facility TAT of less than four weeks. However, studies show that shortening TAT in rural settings is challenging with many rural settings reporting up to 60–90 days caregiver TAT [7, 15]. While active outreach (e.g. [19]) can be used to return HIV-positive results to caregivers and initiate treatment on the infant, turnaround times between the laboratory and the clinic could be more complicated as these require cooperation from multiple levels – the clinic, the laboratory and the transport system. Although SMS printers have been mooted as the solution to shortening the turnaround times between the laboratory and the clinics, their effectiveness depends on receipt of results by someone in the clinic [20].

The observed negative relationship between short EID caregiver TAT and staff nurses could reflect the roles or competence of this staff type. These nurses were those who train for one year; therefore it is likely that they were either not adequately experienced, or permitted to engage in EID activities or supported. Additionally, facilities with largely staff nurses may have with few professional nurses for the number of staff or for the patient population. Consequently, professional nurses in facilities with staff nurses may have been over-burdened by the workload, and thus unable to provide adequate support to staff nurses, who needed advice on how to interpret results or how to contact caregivers. We assume that facilities without staff nurses were mainly staffed by professional nurses who were experienced and trained, explaining our finding of delayed caregiver TAT in facilities with staff nurses compared to facilities without. Professional nurses could not be evaluated in the model because they were present in at least 97% of the facilities. Our results corroborate the need to roll out task shifting with plans for mentoring and support. These findings can also be used to investigate further how the staffing of various types of nurses can be placed at EID facilities in order to improve EID service delivery.

### Limitations

Information on rural versus peri-urban versus urban location of facilities was not available, yet this could have affected TAT [15]. Distances travelled, from facilities via referral laboratories to the nine reference HIV PCR laboratories in the country, were unavailable. All data on EID caregiver TAT and time to transport specimens to the laboratory and return results to the facility were reported by interviewees, and not actually measured, and thus could be an under- or over-estimate. Since interviewees were facility staff, their responses may have been subjective and possibly biased by how they perceived the EID service. However, a complete EID TAT does not depend on the facility alone, but on multiple service providers including the transportation system, the laboratory PCR processing times and the caregiver’s time taken to collect results; thus, there was no convincing reason for interviewees to deliberately bias their response, as the facility was not implicated or judged by the response. Additionally, using the same approach across all sites reduces bias in the analyses and the data becomes comparable to itself. Time that results spent within the facility, i.e., after collecting the blood samples from a baby until it is dispatched from the facility and the time that the HIV results stay in the facility before a caregiver is contacted were not available. The data did not allow us to investigate the required staff nurse-professional nurse ratios needed at EID facilities to maximize the performance of EID service delivery, as we had not identified this as a key research question.

## Conclusion

The results presented in this study are from a situational assessment conducted in 2010, before the introduction of more frequent HIV testing points for infants, and before the implementation of test and treat for life. Thus they serve to raise awareness on the urgency to deal with those factors which delayed EID TAT in 2010, if they are still unchanged to date. The 100% coverage for training professional nurses on infant blood draw for EID observed here as early as 2010, only five years after the launch of the HIV PCR EID program, is encouraging. We show that only 1 in 4 facilities achieved short EID caregiver TAT of ≤3 weeks and that TAT to facilities influenced final EID TATs to caregivers. The negative association with staff nurses also indicates the importance of human resource support in EID service delivery, especially in light of task shifting. Considering the recent introduction of HIV PCR testing at birth, within one week TATs for EID need to be ensured to fast-track ART initiation in HIV infected infants.

## Additional file

Additional file 1: Number of facilities using each transportation system within each province, in 2010. (DOCX 37 kb)

## Data Availability

The data are bound by ethical and legal restrictions. To access the data of the South Africa’s Prevention of Mother to Child Transmission Effective study, investigators who are not part of the study team should submit a concept proposal to AG (South Africa Medical Research Council (MRC), principal investigator), Debra Jackson (University of the Western Cape/UNICEF, principal investigator) and Thu-Ha Dinh, MD, MS (US Centers for Disease Control and Prevention (CDC), principal investigator) for approval. Investigators with an approved concept proposal must apply for guest researcher status to obtain access to a workstation and the data. Additionally, they will need to complete data security and confidentiality training, and to sign data use and non-disclosure agreements. The data are not yet available in a stable public repository. Researchers who meet criteria to access the data should contact the author AG at Ameena.Goga@ mrc.ac.za.

## Abbreviations

aRD: adjusted risk difference
ART: Antiretroviral therapy
EID: Early infant diagnosis
HIV: Human immunodeficiency virus
MTCT: Mother-to-child transmission of HIV
NHLS: National health laboratory services
PCR: Polymerase chain reaction
PMTCT: Prevention of mother-to-child transmission of HIV
SAPMTCTE: South African prevention of mother-to-child transmission of HIV programme evaluation
SOP: Standard operating procedures
TAT: Turnaround times
