# Supplementary material for: Infrastructural and human-resource factors associated with return of infant HIV test results to caregivers: secondary analysis of a nationally representative situational assessment, South Africa, 2010
Source: BMC Infect Dis. 2019 Sep 16;19(Suppl 1):785. doi: 10.1186/s12879-019-4337-0 (PMC6745768; doi:10.1186/s12879-019-4337-0)
Supplement: Supplementary file 1 — Number of facilities using each transportation system within each province, in 2010. (DOCX 37 kb) [file 12879_2019_4337_MOESM1_ESM.docx]

**Additional file 1:** Number of facilities using each transportation system within each province, in 2010

| **Province** | **Transportation system to the laboratory** | | | **Total** |
| --- | --- | --- | --- | --- |
|  | **Provincial** | **NHLS** | **Private** |  |
| Eastern Cape | 1 | 50 | 7 | 64 |
| Free State | 3 | 42 | 21 | 67 |
| Gauteng | 0 | 66 | 2 | 72 |
| KwaZulu Natal | 59 | 3 | 2 | 69 |
| Limpopo | 0 | 24 | 25 | 51 |
| Mpumalanga | 4 | 76 | 0 | 84 |
| Northern Cape | 4 | 20 | 10 | 38 |
| North West | 2 | 29 | 31 | 69 |
| Western Cape | 7 | 37 | 12 | 57 |
| Total | 80 | 347 | 110 | 571 |
